# Complete genome sequences of two *Pseudoalteromonas undina* strains isolated from a marine nematode (Oncholaimidae) collected at Tybee Island

**DOI:** 10.1128/mra.00419-25

**Published:** 2025-07-25

**Authors:** Alejandro De Santiago, Shelby J. Barnes, Tiago J. Pereira, Mirayana Marcelino-Barros, Holly M. Bik, J. Cameron Thrash

**Affiliations:** 1 Institute of Bioinformatics, University of Georgia 1355, Athens, Georgia, USA; 2 Department of Marine Sciences, University of Georgia 1355, Athens, Georgia, USA; 3 Department of Biological Sciences, University of Southern California 118558, Los Angeles, California, USA; University of Maryland School of Medicine, Baltimore, Maryland, USA

## Abstract

*Pseudoalteromonas* is known to form symbiotic relationships with various marine invertebrates, but association with nematodes has not been well-explored. Here, we report the genome sequences of two *Pseudoalteromonas* strains isolated from a predatory marine nematode (Oncholaimidae) collected from Tybee Island, GA, that will facilitate the study of nematode-bacterial interactions.

## ANNOUNCEMENT

*Pseudoalteromonas* bacteria are ubiquitous in marine ecosystems and have been found in both free-living and host-associated contexts, including microbiomes of several marine invertebrate phyla (1–4). Recently, there has been evidence that *Pseudoalteromonas* may occur in the core microbiome of marine nematodes in the family Oncholaimidae (5); however, nematode-associated *Pseudoalteromonas* have eluded whole-genome sequencing.

We isolated two *Pseudoalteromonas* strains from an oncholaimid nematode collected from muddy sediments at Tybee Island, GA, USA (Table 1). Nematodes were isolated from the sediment using a decantation-flotation method (6) and decanted over a 45 µm sieve using sterile artificial seawater (Instant Ocean, Spectrum Brands, Blacksburg, VA). Nematodes were picked under a dissecting microscope (Olympus SZX16, Olympus Corporation, Tokyo, Japan) and rinsed in sterile molecular-grade water, as described (7). We mounted the worms on temporary slides and identified them to the morphospecies level using the appropriate taxonomic keys (8) under a compound microscope (Olympus BX63, Olympus Corporation, Tokyo, Japan). Several nematodes from the same morphospecies (Family: Oncholaimidae) were subsequently transferred to a polymerase chain reaction (PCR) tube with 1 mL SJB1 sterile artificial seawater medium (9) and ground to a slurry using a sterile pipette tip in a biosafety cabinet. The slurry was treated with 0.008% Tween-20, vortexed for 5 min, and centrifuged at 500× g for 55 min at room temperature (RT) to dissociate microbial cells from the nematode biomass. We quantified the supernatant cell concentration using an Accuri C6 Plus flow cytometer (BD Biosciences) and inoculated SJB1 medium for high-throughput dilution culturing and identification as described (10). We grew strains US3C1013 and US3C1004 for sequencing in SJB1 at RT and extracted DNA using our phenol-chloroform method (11).

**TABLE 1:** Sampling location and genome statistics for strains US3C1013 and US3C1004

Parameter	US3C1013	US3C1004
Sample location		
Host	Nematode (Oncholaimidae)	
Location	Tybee Island, GA	
Latitude	32.0157	
Longitude	−80.8911	
Depth	0.5 m	
Sample collection	December 2020	
Isolation experiment	March 2021	
Genome summary		
Taxonomy (GTDB-tk)	*Pseudoalteromonas undina*	*Pseudoalteromonas undina*
Reference (GTDB-tk)	GCF_000238275.3	GCF_000238275.3
Genome size (bps)	4,118,019 (3,320,003 + 788,016)	4,107,746 (3,319,730 + 788,016)
Number of replicons	2	2
Circularized?	Yes	Yes
GC content	40%	40%
Number coding sequences	3,774	3,773
Completeness (CheckM)	100%	100%
Contamination (CheckM)	0.71%	0.71%
Coding density (CheckM)	0.883	0.884
Coverage (Flye)	100×	83×
Num read pairs Illumina	1,773,627	2,582,410
Num reads Nanopore/N50	226,322/35,802	262,460/40,362
Assembly accession	GCA_048401115.1	GCA_048401065.1
SRA accession Illumina	SRX27591692	SRX27591694
SRA accession Nanopore	SRX27591693	SRX27591695

DNA from the same extractions was prepared for hybrid Oxford Nanopore and Illumina sequencing with an Oxford Nanopore Technologies native barcoding kit (#SQK-NBD114) and an Illumina DNA Prep tagmentation kit (#20060059) at SeqCoast Genomics. The Long Fragment Buffer was used to promote longer read lengths. No physical size selection was done. Sequencing was performed on an Illumina NextSeq2000 using a 300-cycle flow cell kit, producing 2 × 150 bp paired-end reads. A 1%–2% PhiX control was spiked into the run to support optimal base calling. Nanopore sequencing was completed using a FLO-PRO114M version R10.4.1 flow cell (translocation speed 400 bps) on a PromethION 2 Solo sequencer. Base-calling was performed using the super-accurate model with barcode trimming enabled with MinKNOW v24.02.16, Dorado v7.3.11 (Oxford Nanopore). Reads were demultiplexed and trimmed using DRAGEN v3.10.2. We assembled contigs using Flye v2.9.1-b1780 (12) with four iterations and polished with short reads using Polypolish v0.5.0 (13). This generated circularized genomes, including one primary chromosome and one extrachromosomal element in each. No separate rotations of the genomes were done. Percent completeness and contamination were assessed using CheckM2 v1.0.0 *predict* (14). Genomes were classified as *Pseudoalteromonas undina* using GTDB-tk v2.1.1 *classify_wf* (15) and annotated via the NCBI Prokaryotic Genome Annotation Pipeline (16). Default settings were used for all software unless otherwise stated. Genome statistics are summarized in Table 1.

## Data Availability

Sequencing reads and genomes (accession numbers in Table 1) are available at NCBI under BioProject number PRJNA1219708.
